# Attitudes towards the neurological examination in an unwell neonate: a mixed methods approach

**DOI:** 10.1186/s12887-022-03616-4

**Published:** 2022-09-23

**Authors:** Ala Fadilah, Quentin Clare, Anthony Richard Hart

**Affiliations:** Department of Paediatric and Perinatal Neurology, Sheffield Children’s Hospital NHS Foundation Trust, Ryegate Children’s Centre, Tapton Crescent Road, Sheffield, S10 5DD UK

**Keywords:** Infant, Newborn, Neurology, Neurologic examination, Diagnosis differential, Education, Medical, Hypoxia-ischaemic, Brain

## Abstract

**Background:**

The neurological examination of an unwell neonate can aid management, such as deciding if hypothermia treatment is warranted in hypoxic ischaemic encephalopathy or directing investigations in hypotonic neonates. Current standardised examinations are not designed for unwell or ventilated neonates, and it is unclear how confident paediatricians feel about the examination or what aspects they perform.

**Aim:**

This study aimed to review the confidence of UK paediatricians on the neurological examination in unwell neonates, describe their attitudes towards it, and determine what could improve practice.

**Methods:**

An explanatory sequential mixed methods approach (QUAN → QUAL) with equal weighting between stages. A survey on attitudes to the neonatal neurological examination was sent to all UK neonatal units and members of the British Paediatric Neurology Association. Volunteers were sought for semi-structured interviews. Thematic analysis was used to interpret qualitative data, which was triangulated with quantitative questionnaire data.

**Results:**

One hundred ninety-three surveys were returned, 31.0% from neonatologists, 9.3% paediatric neurologist. The median range for confidence was 4 (IQR3-5). Twenty-three interviews occurred. Thematic analysis revealed three themes: “Current culture on neonatal units”, “ Practicalities of the neurological examination in unwell neonates”, and “Changing the culture”. Most interviewees did not feel confident performing or interpreting the neurological examination in unwell neonates. Many units had a culture of seeing it as low priority, did not see its relevance in the acute management of unwell neonates. A few interviewees worked in units with a positive culture towards the neurological examination who used adapted standardised examinations and provided training. 72% of questionnaire responders wanted a new standardised neurological examination designed for the unwell neonate, which should be short, utilise pictures like the Hammersmith Neonatal Neurological Examination, contain an assessment of consciousness, be developmentally appropriate and achievable in unwell, ventilated neonates, be accompanied by a schematic to aid interpretation, and for greater training and assessments of competence.

**Conclusions:**

There are barriers preventing paediatricians being able to perform a neurological examination in unwell neonates, and a culture of neurophobia is common. A new standardised examination is needed, alongside aids to interpretation, training, and assessment.

**Supplementary Information:**

The online version contains supplementary material available at 10.1186/s12887-022-03616-4.

## Introduction

After assessment and resuscitation, the starting point for any patient evaluation is history and examination. The examination of the neurological system is complex in children, requiring adaptation for their developmental abilities and behaviour [1]. The results can direct treatment; for example, in perinatal hypoxic ischaemic encephalopathy (HIE) the neurological examination determines whether hypothermia treatment is warranted or not [2, 3]. Following commencement of hypothermia, it also helps monitor change and informs prognostication. Outside of HIE, it determines the anatomical site of abnormal signs and supports formulation of differential diagnoses and management plans.

In the UK, postgraduate education begins with two years foundation level training, incorporating a variety of adult specialities and, rarely, paediatric posts. Paediatric training begins thereafter. The first 5 years are focussed on attaining generic core competencies, encompassing 3 years as a junior trainee (ST1-3) and 2 years as a senior trainee (ST4-5), with at least one year in tertiary neonatal services. During core training, paediatricians acquire competencies in examination in a variety of specialities, including general paediatrics, emergency medicine, neurology, community / neurodisability paediatrics, intensive care, and so forth. Thereafter, a trainee continues through 2–3 years of training in general paediatrics (ST6-8). Thus, all UK paediatric trainees should receive the same training on the neurological examination of a neonate, although there is no standardisation to this training.

There is little data on how confident and competent paediatricians are at performing the neurological examination in an unwell neonate. Previous work in our region showed trainees felt confident in perinatal HIE and reported they documented it thoroughly [4]. This was inconsistent with our experience. When asked to list what aspects of the examination trainees performed, they listed only cursory or limited aspects of the examination. At the time, reviewers concluded our results reflected poor training in our geographic area, but our experience was it reflected an attitudinal problem across the country, if not the world. If true, a thorough education programme is needed to improve patient examination and assessment, which could have benefits for patient care. This cannot be created without knowing what training is happening currently, what challenges paediatricians face with the neonatal neurological examination, and what tools are needed to assist them.

This study aimed to review UK paediatricians’ confidence about the neonatal neurological examination, describe their attitudes towards it, the challenges they face, and to ascertain what would improve practice.

## Methods

We adopted an explanatory sequential mixed methods approach in two distinct phases (QUAN → QUAL) [5]. A questionnaire (Phase I) was distributed to examine health care professionals’ confidence in the neonatal neurological examination, followed by qualitative interviews (Phase II) to describe the reasons for these results. Equal weighting was given to both phases.

### Phase I

In England, several specialities can be involved in the assessment of an unwell term neonate: neonatologists on Level 3 neonatal units, paediatricians on Level 1 and 2 neonatal units, paediatricians receiving referrals from the emergency room and primary care, and paediatric neurologists. Neonatologists and paediatricians are more likely to review acute emergencies where the diagnosis is clear, such as perinatal HIE, whilst neurologists are more likely to be determining the cause and severity of a neonate’s neurological state. Because the initial training of all paediatricians is the same, we hypothesised there would be homogeneity in views on training and performing the neonatal neurological examination. We therefore aimed to sample views from all these paediatricians.

We designed a questionnaire in paper and electronic versions (Supplementary material 1) based on our previous experience [4], and trialled it prior to use. The trial included asking 6 paediatricians (2 consultant paediatric neurologists, 1 speciality doctor in paediatric neurology and 3 paediatric trainees) to complete it either in paper or online format. They were asked to comment on the length of the questionnaire, whether the questions would give us information we wanted, and to identify any ambiguous or confusing language / questions. We rewrote problematic questions and improved the layout of the questionnaire based on these comments. The description of “sick” or “unwell” was outlined as a baby with illness like encephalopathy, weakness, or those who were ventilated / had umbilical or other lines in situ. This encompassed a large number of conditions depending on the specialist interest of the responder. Participants were asked to focus on the term neonate when answering, although they could also comment on preterm examination if they wanted. The questionnaire including questions with Likert scores ranging from 0 (never, not well / easy, or low confidence) to 6 (always, very well / easy, or high confidence) covering how often paediatricians perform the neurological examination, how easy they find performing it and its constituent parts, how well they interpreted its results and used them to make a management plan, and whether they used standardised neonatal neurological examinations or not. A free text box allowed responders to explain what challenges they faced when performing the neonatal neurological examination.

One hundred ninety-six Neonatal units were identified from a national website (ukntg.net/uk-neonatal-units). The clinical lead was asked if they and other staff members would complete the questionnaire. Members of the British Paediatric Neurology Association (BPNA) were asked to complete it via a monthly e-newsletter. Responders were asked to advertise the questionnaire to others. Responders were those who performed the neurological examination in clinical practice. This included Paediatricians and Advanced Neonatal Nurse Practitioners (ANNPs). ANNPs are highly trained nurses with additional qualifications and training, whose daily work is the same as paediatric trainees. We report frequencies, percentages, median, and interquartile ranges of responses. The free text comments were grouped into natural categories, and the frequency of responses falling within each category reported.

Connection of the two phases: the last question of the questionnaire asked for volunteers for a qualitative interview. Our inclusion criteria were paediatric doctors who assessed unwell neonates. We did not recruit Advanced Nurse Practitioners to qualitative interviews because their response rate to the questionnaires was low and recruitment would have been hard. We were also aware their training programme differed from doctors’, which may have led to greater variation in views and the need for a larger sample size. From the list of volunteers, a convenience sample was used to make recruitment easier; however, we selected a spread of grades of doctor and specialities. We did not expect gender to influence answers, but our previous experience was that male doctors were more likely to arrange interviews [6, 7], so we ensured a gender balance in this cohort. We selected interviewees from a wide geographical area of England to avoid results reflecting training issues specific to a particular region. No more than 3 participants were recruited from any single centre. Once we had identified initial participants from the questionnaire, we used a snowball technique, i.e. asking participants to recommend further potential participants, where specific demographics were underrepresented. The purpose of the interviews was to describe attitudes towards the neurological examination in the unwell neonate, how interviewees were trained, and how they reached confidence. We asked interviewees to focus particularly on the term neonate. The topic guide (Supplementary material 2) was influenced by the questionnaire and was trialled in 3 volunteers, and the questions and their order were changed to improve the interviews.

### Phase II

A qualitative descriptive methodology was used. A single interviewer performed the semi-structured interviews (AF). The interviewer was a female paediatric neurology trainee. The interviewer may have known the interviewee previously and had preconceived views that could lead to bias. We reduced this risk through training and by ensuring the topic guide contained open questions. Interviews occurred at a time and location of the participants’ choice and in person/face-to-face, where possible. During the COVID-19 pandemic, interviews were performed virtually via video link. Written informed consent was obtained. The interviews were recorded digitally, transcribed verbatim by a medical secretary, and anonymised, with names replaced by participant numbers and working or training locations exchanged for “X”. One of the research team listened to the recorded interviews whilst reviewing the transcript to check it for accuracy. The researcher corrected typing errors. One participant, for whom English was not their first language, asked to review their transcript after it had been reviewed by the research team to ensure it accurately reflected their views.

Thematic analysis was performed as per Braun and Clarke (2006) [8], including familiarisation of data, coding using an inductive approach by two researchers (QC and ARH), review of initial codes, agreement on a coding structure, reflexive changes to coding structure as more data was analysed, and identification of a thematic structure. Themes were developed using an iterative process. With relation to researcher reflexivity, ARH was a consultant paediatric neurologist, who was aware he had preconceived ideas about the neonatal neurological examination in clinical practice. He ensured he questioned his assumptions regularly during the analytical process. QC was a non-medical research fellow with training in qualitative research, who was previously aware of the challenges faced in neonatal care from discussions with health care professionals. Both researchers reviewed and discussed their coding structure together repeatedly during data analysis and refined them after discussion. The themes were formulated independently, and then discussed and revised, with both researchers able to challenge the other’s assumptions.

We reached data saturation at 20 participants. Data saturation was defined as occurring when additional data did not significantly change or refine the coding structure, nor thematic framework, i.e.thematic exhaustion occurred [9, 10]. We recruited a further 3 to ensure no new codes or themes arose, and to ensure a fair balance of interviewee characteristics. The concept of data saturation is controversial, along with how many participants are needed to reach this [11, 12]. We thought our 23 participants was sufficient because our study had relatively narrow aims, i.e. to determine views on performing the neonatal neurological examination, and participants’ training of the neonatal neurological examination would have been similar. Furthermore, our interviews were relatively long, ranging from 55 min to over 120 min, contained rich in-depth data, and the same interview schedule was used for all interviews. NVivo for Mac version 12 (QSR International PTY Ltd, 2018) was used for analysis. The results of the quantitative and qualitative phases were integrated by determining the messages provided by both sections of the study and identifying areas in which they agreed or disagreed. Where the messages differed, we looked at our qualitative data to determine if there was an explanation. We also studied the results of our previous quantitative questionnaire [4], and other previously published data in this field to explain our results and to inform our discussion. Ethical approval was obtained from the Nottingham 1 Research Ethics Committee (IRAS 259,148) and informed consent obtained from all interviewees.

## Results

### Quantitative data

One hundred ninety-three questionnaires were returned with no duplicates. Responders worked in 60 units across the UK, although 98 responders did not report location. Multiple responses from single units were received, with the largest being our own: 6 from our Children’s Hospital and 3 from the Maternity Hospital. 60/193 (31.0%) responders worked in neonatology, 111 (57.6%) in paediatric specialities other than neurology, 18 (9.3%) in paediatric neurology, 4 (2.1%) in Paediatric Emergency Medicine / Anaesthesia. Ninety-two (47.7%) were consultants, 57 (29.5%) ST4-8 trainees, 25 (13.0%) ST1-3 trainees, 8 (4.1%) Advanced Neonatal Nurse Practitioners, and 11 (5.7%) other grades.

The results to questions on general attitude and practice relating to the neurological examination in the unwell neonate are summarised in table one. Some respondents did not answer all of the questions (see Tables 1 and 2). Variation in practice was noted with reference to the neurological examination in an unwell neonate: trainees and Advanced Neonatal Nurse Practitioners (ANNPs) reported they performed a neurological examination in around half of neonates, and consultants in most. Within the consultant responses ranges, some reported they hardly ever performed the examination. The median range for confidence was high (Table 1), although a wide range was seen. Responders reported that a high-quality documentation of a neurological examination was found in the medical records of an unwell term neonate around half of the time, with the range of scores extending to ‘never’. Neurologists scored this lower. A small proportion of responders routinely used a standardised neurological examination, including the Hammersmith Neonatal Neurological Examination (HNNE), to assess unwell neonates.

**Table 1:** Responses to the survey questions on attitudes to the neurological examination in an unwell neonate

Question	Possible Answers	Whole cohort	By Grade				By speciality			
			*ST1-3*	*ST4-8 and other*	*Consultant*	*ANNP*	*Tertiary neonatology*	Paediatrics*		*Paediatric Neurology*
**How often do you perform a neurological examination in an unwell neonate?**	Scale 0–6, where 0 = never; 3 = about half the time; 6 = all the time	4 (IQR 3–6; range 0–6)	3 (IQR 2–4; range 0–6)	4 (IQR 3–6; range 0–6)	5 (IQR 3–6; range 1–6)	3.5 (IQR 3–5.25; range 2–6)	5 (IQR 3.25–6; range 1–6)		4 (IQR 3–6; range 0–6)	3 (IQR 2–5.75; range 1–6)
**How confident do you feel performing a neonatal neurological examination?**	Scale 0–6, where 0 = not at all confident; 3 = somewhat confident; 6 = completely confident	4 (IQR 3–5; range 0–6)	3 (IQR 2–4; range 0–5)	4 (IQR 3–5; range 0–6)	5 (IQR 4–5; range 2–6)	3.5 (IQR 2–4; range 1–5)	4 (IQR 4–5; range 1–6)		4 (IQR 3–5; range 0–6)	5 (IQR 4–5; range 3–6)
**How confident do you feel interpreting the results of a neonatal neurological examination i.e. establishing whether normal or abnormal; determining anatomical site of any abnormality**		4 (IQR 3–5; range 0–6)	2 (IQR 1–3.25; range 0–5)	3 (IQR 3–4; range 0–6)	4.5 (IQR 4–5; range 2–6)	3 (IQR 1.75–4.25; range 0–5)	4 (IQR 3–5; range 0–6)		3 (IQR 3–5; range 0–6)	5 (IQR 4–5; range 2–6)
**How confident do you feel using results of neonatal neurological examination to make a management plan**		4 (IQR 3–5; range 0–6)	2 (IQR 1–3.25; range 0–6)	4 (IQR 2.75–5; range 1–6)	5 (IQR 4–5; range 2–6)	3 (IQR 2.75–4.25; range 0–5)	4 (IQR 4–5; range 0–6)		4 (IQR 2–5; range 0–6)	5 (IQR 4–5; range 2–6)
**How confident do you feel using results of neonatal neurological to form a prognosis**		3 (IQR 2–4; range 0–6)	2 (IQR 0.75–3; range 0–5)	3 (IQR 2–4; range 0–5)	4 (IQR 3–5; range 0–6)	2.5 (IQR 1.5–3.25; range 0–5)	4 (IQR 2.5–5; range 0–6)		3 (IQR 2–4; range 0–6)	4 (IQR 3–4; range 2–6)
**How often do you find a detailed, good quality neurological examination in the notes of a baby with a condition like encephalopathy or weakness?**	Scale 0–6, where 0 = never; 3 = about half the time; 6 = every time	3 (IQR 2–4; range 0–6)	2 (IQR 1–3.25; range 0–6)	3 (IQR 1.75–4; range 0–6)	3 (IQR 2–4; range 1–6)	2 (IQR 1–2.25; range 0–5)	3 (IQR 2–4; range 0–6)		3 (IQR 2–4; range 0–6)	1.5 (IQR 1–2; range 1–4)
**How well do you think the trainees in your department perform the neonatal neurological examination in an unwell baby?**	Scale 0–6, where 0 = not at all well; 3 = somewhat well; 6 = completely well	3 (IQR 2–3; range 0–6)	2.5 (IQR 2–3; range 0–6)	3 (IQR 2–4; range 0–5)	3 (IQR 2–3; range 0–5)	2.5 (IQR 1–3.25; range 0–4)	3 (IQR 2–4; range 0–5)		3 (IQR 2–3; range 0–6)	3 (IQR 2–3; range 1–5)
**How often do you use the classical paediatric neurological examination, adapted for neonates**	I have never used it in a neonate	30/168 (17.9%)	6/22 (27.3%)	9/58 (15.5%)	14/83 (16.9%)	1/5 (20%)	6/52 (11.5%)		23/101 (22.8%)	1/15 (6.7%)
	I used this in the past, but I do not use it routinely now	20/168 (11.9%)	3/22 (13.6%)	8/58 (13.8%)	9/83 (10.8%)	0/5 (0%)	11/52 (21.2%)		9/101 (8.9%)	0/15 (0%)
	I use this in specific cases only	33/168 (19.6%)	5/22 (22.7%)	10/58 (17.2%)	18/83 (21.7)	0/5 (0%)	8/52 (15.4%)		22/101 (21.8%)	3/15 (20.0%)
	I use this routinely in most neonates	85/168 (50.6%)	8/22 (36.4%)	31/58 (53.5%)	42/83 (50.6%)	4/5 (80%)	27/52 (51.9%)		47/101 (46.5%)	11/15 (73.3%)
**Hammersmith Neonatal Neurological Examination**	I have never used it in a neonate	102/168 (60.7%)	18/22 (81.8%)	37/59 (62.7%)	44/82 (53.7%)	3/5 (60.0%)	25/52 (48.1%)		71/100 (71.0%)	6/16 (37.5%)
	I used this in the past, but I do not use it routinely now	27/168 (16.1%)	2/22 (9.1%)	7/59 (11.9%)	17/82 (20.7%)	1/5 (20.0%)	8/52 (15.4%)		16/100 (16.0%)	3/16 (18.8%)
	I use this in specific cases only	32/168 (19.0%)	2/22 (9.1%)	13/59 (22.0%)	16/82 (19.5%)	1/5 (20.0%)	16/52 (30.7%)		10/100 (10.0%)	6/16 (37.5%)
	I use this routinely in most neonates	7/168 (4.2%)	0/22 (0%)	2/59 (3.4%)	5/82 (6.1%)	0/5 (0%)	3/52 (5.8%)		3/100 (3.0%)	1/16 (6.2%)
**Adapted (i.e. you add or omit certain bits) Hammersmith Neonatal Neurological Examination**	I have never used it in a neonate	101/165 (61.2%)	15/22 (68.2%)	40/59 (67.8%)	43/79 (54.4%)	3/5 (60.0%)	25/52 (48.1%)		71/99 (71.7%)	5/14 (35.7%)
	I used this in the past, but I do not use it routinely now	14/165 (8.5%)	1/22 (4.5%)	4/59 (6.8%)	9/79 (11.4%)	0/5 (0%)	3/52 (5.8%)		10/99 (10.1%)	1/14 (7.2%)
	I use this in specific cases only	35/165 (21.2%)	5/22 (22.8%)	9/59 (15.3%)	20/79 (25.3%)	1/5 (20.0%)	19/52 (36.5%)		11/99 (11.1%)	5/14 (35.7%)
	I use this routinely in most neonates	15/165 (9.1%)	1/22 (4.5%)	6/59 (10.1%)	7/79 (8.9%)	1/5 (20.0%)	5/52 (9.6%)		7/99 (7.1%)	3/14 (21.4%)
**Brazelton Neonatal Behavioural Assessment Scale**	I have never used it in a neonate	148/163 (90.8%)	22/22 (100%)	54/59 (91.5%)	67/77 (87.0%)	5/5 (100%)	46/52 (88.5%)		89/97 (91.8%)	13/14 (92.9%)
	I used this in the past, but I do not use it routinely now	7/163 (4.3%)	0/22 (0%)	1/59 (1.7%)	6/77 (7.8%)	0/5 (0%)	3/52 (5.8%)		4/97 (4.1%)	0/14 (0%)
	I use this in specific cases only	6/163 (3.7%0	0/22 (0%)	3/59 (5.1%)	3/77 (3.9%)	0/5 (0%)	2/52 (3.8%)		3/97 (3.1%)	1/14 (7.1%)
	I use this routinely in most neonates	2/163 (1.2%)	0/22 (0%)	1/59 (1.7%)	1/77 (1.3%)	0/5 (0%)	1/52 (1.9%)		1/97 (1.0%)	0/14 (0%)
**Amiel-Tison Neurologic Assessment**	I have never used it in a neonate	140/164 (85.3%)	22/22 (100%)	52/59 (88.1%)	64/78 (82.1%)	2/5 (40.0%)	42/52 (80.8%)		86/97 (88.6%)	12/15 (80.0%)
	I used this in the past, but I do not use it routinely now	8/164 (4.9%)	0/22 (0%)	3/59 (5.1%)	5/78 (6.4%)	0/5 (0%)	2/52 (40.0%)		5/97 (5.2%)	1/15 (6.7%)
	I use this in specific cases only	10/164 (6.1%)	0/22 (0%)	2/59 (3.4%)	6/78 (7.7%)	2/5 (40.0%)	5/52 (9.6%)		3/97 (3.1%)	2/15 (13.3%)
	I use this routinely in most neonates	6/164 (3.7%)	0/22 (0%)	2/59 (3.4%)	3/78 (3.8%)	1/5 (20.0%)	3/52 (5.8%)		3/97 (3.1%)	0/15 (0%)

^*^General paediatrics and responders from other specialities, such as Emergency Paediatrics, Gastroenterology, and so forth

**Table 2:** Responses to the survey questions confidence of specific aspects of the neurological examination in unwell neonates

Question		Possible Answers	Whole cohort	By Grade				By speciality		
				*ST1-3*	*ST4-8 and other*	*Consultant*	*ANNP*	*Tertiary neonatology*	Paediatrics*	*Paediatric Neurology*
**How easy is it to assess the following aspects of the neurological examination of a sick neonate?**	Conscious level	Scale 0–6, where 0 = not at all easy; 3 = somewhat easy; 6 = completely easy	4 (IQR 2.75–5; range 0–6)	3 (IQR 2–4; range 0–6)	4 (IQR 3–5; range 0–6)	4 (IQR 3–5; range 0–6)	1 (IQR 1–5); range 1–6)	4 (IQR 3–5; range 0–6)	4 (IQR 2–5; range 0–6)	3 (IQR 3–4; range 1–6)
	Quantity of spontaneous movements		4 (IQR 3–5; range 0–6)	4 (IQR 3–5; range 1–6)	4 (IQR 3–5; range 0–6)	4 (IQR 3–5; range 0–6)	3 (IQR 1–5; range 1–6)	4 (IQR 3–5; range 1–6)	4 (IQR 3–5; range 0–6)	4 (IQR 3–5; range 1–5)
	Quality of movements		4 (IQR 3–4; range 0–6)	3.5 (IQR 2–5; range 0–6)	4 (IQR 2.5–4; range 0–6)	4 (IQR 3–4; range 0–6)	4 (IQR 2–4; range 1–6)	4 (IQR 3–4; range 1–6)	4 (IQR 2.75–5; range 0–6)	4 (IQR 3–4; range 2–5)
	Limb tone		4 (IQR 3–5; range 0–6)	4 (IQR 3.25–5; range 0–6)	4 (IQR 3–5; range 0–6)	4 (IQR 3–5; range 1–6)	4 (IQR 3–4; range 2–6)	4 (IQR 3–5; range 2–6)	4 (IQR 3–5; range 0–6)	3 (IQR 3–5; range 2–6)
	Truncal tone		4 (IQR 2–5; range 0–6)	3 (IQR 2–4.75; range 0–6)	4 (IQR 2–5; range 0–6)	4 (IQR 2–4; range 1–6)	2 (IQR 2–4; range 2–4)	3.5 (IQR 2–4.25; range 1–6)	4 (IQR 2–5; range 0–6)	3.5 (IQR 3–5; range 1–6)
	Muscle power		3 (IQR 2–4; range 0–6)	3 (IQR 2–3.75; range 0–6)	3 (IQR 2–4; range 0–6)	3 (IQR 2–4; range 0–6)	2 (IQR 2–3; range 2–4)	3 (IQR 2–4; range 0–6)	3 (IQR 2–4; range 0–6)	3 (IQR 2–4; range 1–5)
	Deep tendon reflexes		3 (IQR 2–4; range 0–6)	2 (IQR 1.25–3; range 0–6)	3 (IQR 2–4; range 0–6)	3 (IQR 2–4; range 0–6)	1 (IQR 1–1; range 0–1)	3 (IQR 1–4; range 0–6)	3 (IQR 2–4; range 0–6)	4 (IQR 3–5; range 1–6)
	Primitive reflexes		4 (IQR 3–5; range 0–6)	4 (IQR 3–5; range 0–6)	5 (IQR 3–5; range 0–6)	4 (IQR 3–5; range 0–6)	3 (IQR 3–3; range 2–6)	4 (IQR 3–5; range 1–6)	4 (IQR 3–5; range 0–6)	5 (IQR 3.5–5; range 3–6)
	Cranial nerve function		2 (IQR 1–3; range 0–6)	2 (IQR 1–3; range 0–4)	2 (IQR 0–3; range 0–6)	2 (IQR 1–3; range 0–6)	1 (IQR 0–1; range 0–2)	2 (IQR 1–3; range 0–4)	2 (IQR 0–3; range 0–6)	2 (IQR 2–3; range 0–5)
	Anterior fontanelle		6 (IQR 5–6; range 2–6)	6 (IQR 4–6; range 2–6)	6 (IQR 5–6; range 3–6)	6 (IQR 5–6; range 3–6)	6 (IQR 4–6; range 3–6)	6 (IQR 5–6; range 3–6)	6 (IQR 5–6; range 2–6)	5.5 (IQR 5–6; range 4–6)
	Pupillary responses		5 (IQR 4–5.5; range 0–6)	5 (IQR 3–6; range 1–6)	5 (IQR 4–5; range 0–6)	5 (IQR 4–5; range 0–6)	4 (IQR 4–6; range 2–6)	5 (IQR 4–6; range 2–6)	5 (IQR 4–5; range 0–6)	5 (IQR 3.75–6; range 2–6)
	Visual ability		3 (IQR 1–4; range 0–6)	3 (IQR 2–4; range 0–6)	3 (IQR 2–4; range 0–6)	2.5 (IQR 1–4; range 0–6)	0 (IQR 0–1; range 0–5)	2 (IQR 1–3.5; range 0–6)	3 (IQR 1–4; range 0–6)	4 (IQR 3–5; range 0–6)
	Eye movements		3 (IQR 2–4; range 0–6)	2 (IQR 1–3; range 0–6)	3 (IQR 2–4; range 0–6)	3 (IQR 2–4; range 0–6)	1 (IQR 0–2; range 0–6)	2 (IQR 1.75–4; range 0–6)	2 (IQR 2–4; range 0–6)	3 (IQR 2–4; range 0–6)
	Facial expression		3 (IQR 2–4; range 0–6)	3 (IQR 2.25–4; range 0–6)	3 (IQR 2–4; range 0–6)	3 (IQR 2–4; range 0–6)	2 (IQR 2–4; range 1–6)	3 (IQR 2–4; range 0–6)	3 (IQR 2–4; range 0–6)	3.5 (IQR 2.75–4; range 1–6)
	Fundal examination		1 (IQR 0–3; range 0–6)	0.5 (IQR 0–2; range 0–4)	1 (IQR 0–2.5; range 0–5)	2 (IQR 0–3; range 0–6)	1 (IQR 1–2; range 0–3)	2 (IQR 0–3; range 0–6)	1 (IQR 0–2; range 0–5)	2 (IQR 0.75–3; range 0–5)
	Gag		4 (IQR 3–5; range 0–6)	3.5 (IQR 2–5; range 0–6)	4 (IQR 3–5; range 0–6)	5 (IQR 4–5; range 0–6)	5 (IQR 3–6; range 2–6)	5 (IQR 4.5–6; range 2–6)	4 (IQR 2–5; range 0–6)	4 (IQR 4–5.5; range 0–6)
	Suck		5 (IQR 4–6; range 2–6)	5.5 (IQR 4–6; range 2–6)	5 (IQR 4–6; range 2–6)	5 (IQR 5–6; range 2–6)	5 (IQR 3–6; range 2–6)	5 (IQR 5–6; range 2–6)	5 (IQR 4–6; range 2–6)	5 (IQR 4–6; range 3–6)

*General paediatrics and responders from other specialities, such as Emergency Paediatrics, Gastroenterology, and so forth

Table two shows data on how easy responders thought individual aspects of the neurological examination in an unwell neonate were to perform. The following aspects were rated as a median of 5 or more by the whole cohort, indicating ease of examination: anterior fontanelle, pupillary size, and suck. The aspects considered the hardest to perform, defined as a median score of 2 or less were: fundal examination and cranial nerve function. Despite rating cranial nerve function as being hard, responders rated most individual aspects of cranial nerve function as being easier, including pupillary responses, vision, eye movements, facial expression, suck, and gag.

Responders were asked to document the challenges they faced when performing the examination in an unwell neonate. The most common answer was the effect of sedation and muscle relaxants on clinical signs (*n* = 61), followed the physical barrier provided by lines, ventilation tubes, incubators and other equipment and concern about dislodging them (*n* = 56). The other challenges were grouped into natural categories and the most frequent were:

- Cardiovascular instability or the baby needing ‘minimal handling’ (*n* = 25)
- Not knowing how to interpret the findings (20)
- Difficulty eliciting the abnormal signs (13)
- Absent or poor training on the neonatal neurological examination (11)
- Time constraints / the examination takes too long (12)
- A lack of experience or opportunity to practice the examination (12)
- Not knowing what to do (11)
- Lack of an appropriate standardised neonatal neurological examination for unwell neonates (10)
- Understanding normal from abnormal findings at different gestations in preterm neonates (10)
- Lack of confidence (9)
- Difficulty determining whether abnormal signs are a result of a primary neurological disorder or multisystem illness, like septicaemia (9)
- Stabilisation / other procedures take priority (8)
- Subjectivity and reproducibility (7)
- Equipment is unavailable, especially tendon hammers (7).

When responders were asked whether a new standardised neurological examination specifically designed for unwell neonates would be useful, 124/172 (72.1%) agreed, 39/172 (22.7%) were unsure and 9/172 (5.2%) thought it would not be useful.

### Qualitative data

**Fig. 1:**
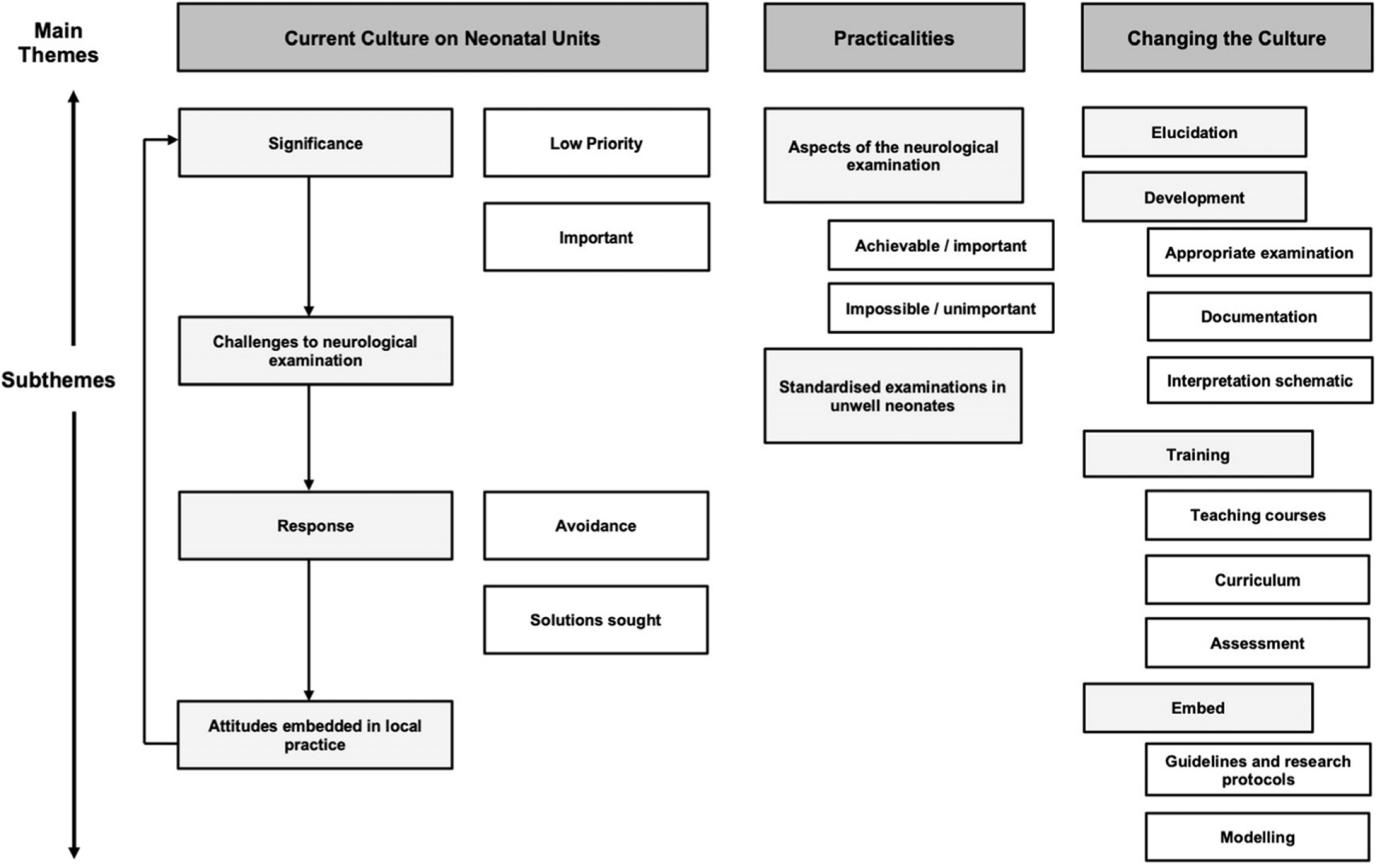
Summary of main themes and subthemes

Twenty-three interviews were performed. Nine volunteers via questionnaire did not respond to invitations to arrange an interview, and 1 was unavailable. Two identified via snowballing did not respond to invitations and 1 cancelled owing to changing work patterns. No repeat interviews occurred. Fourteen interviewees were consultants, 7 ST4-8 trainees, and 2 other grades of doctors. Ten worked in neonatology, 7 in paediatrics, and 6 in paediatric neurology. Twelve were female. Six worked in Yorkshire and the Humber, 3 in North England, 3 East of England, 6 the Midlands, and 5 in London. The length of interviews ranged from 53–122 min, median 84 min. Three themes emerged (Fig. 1):

1. *“Current culture on neonatal units”*
2. *“Practicalities of the neurological examination in unwell term neonates”.*
3. *“Changing the culture”*

### Theme 1: Current culture on neonatal units

Illustrative quotations are shown in Table 3. Neonatologists viewed the neurological examination through the lens of HIE, whilst neurologists focussed on the aetiology of neurological signs. Two quotations highlighted the perceived purpose of the neurological examination: *“the history gives you the mechanism [of injury], and the examination gives you the site”* and “*The history tells you more than the examination. The examination is just to grade the severity of it”*. The value of the examination formed the first subtheme, with two cultures adopted: low priority and important.

**Table 3:** Illustrative quotations for Theme 1—current culture on neonatal units

Subtheme	Quotation	Interviewee (No., Grade, Speciality, Gender)
***Significance: Low Priority***		
Obvious who needs hypothermia treatment	I suppose, often, you put it down to a gut-feeling, um, without sort of doing a systematic, structured examination	2, Tr, PNeurol, M
Emergency and procedures are prioritised	Priority is always, and rightfully so, “ABC”—they are the priority. But, yeah, a neuro exam, a proper neuro exam, is definitely taken back-seat to the tasks, the ‘fun’. Neonatal, task-orientated trainees want to do lines and tubes and X-rays and all that exciting stuff, rather than wash your hands and lay your hand on the baby and actually examine the baby	4, Tr, Neo, M
Other systems are seen as more important	Because people find [other systems] life-threatening that they can support immediately, whereas the bit of the neurological examination that you need to support is the respiratory component….So, it’s not a system that you immediately support	3, C, Neo, M
Don’t see usefulness of neurological examination	I’m not sure that I get any useful information about… when I assess sick babies from the neurological point of view. And I’m not sure how useful that is in the long term. That’s probably why	10, C, Neo, M
	if you don’t think it’s important, then you’re not going to do it. But sometimes you don’t think it’s important because you don’t understand why it’s important,	21, Tr, Paed, F
Neurological examination is time consuming	In my head there’s this, er, idea that neurological examination is really time-consuming	18, Tr, Paed, F
Cannot examine after muscle relaxation	It’s only when you sort of get to a point of stabilisation, erm, and after the stabilisation has occurred, that people remember, ‘maybe we should do a neurological examination’, which of course is difficult to do if they’ve been muscle relaxed	11, C, Paed, M
Relying on other investigations rather than neurological examination	When it comes to neurological assessment and management, we get driven by tests rather than clinical assessment. This my feeling	16, C, Paed, M
***Significance: Important***		
Important	It is an absolutely essential part of the examination, so it needs to be done. It is invaluable information for err very important decision making	9, C, Neo, F
Justifying decisions about hypothermia	The documentation is for someone else to understand why I did what I did, which is really important for me. It’s not because ten years later lawyers will sue me	5, C, Neo, M
Importance of monitoring change over time	Although there are many ways of assessing a neurological system of a newborn, what is important is the trend. We look at the change over time in a sequence of neurological assessments to be able to determine the status and the prognosis	14, C, Neo, F
The neurological examination is fun!	I think examination skills in people need to be inspired by the senior doctors: to see the importance and the pleasure you can get from being a Sherlock Holmes and looking out… looking out for signs and making a clever diagnosis. It can be a motivation, can’t it?	12, C, PNeurol, M
The neurological examination is quick	They don’t realise how quick it is, if you just learn to do it properly it’s very quick	14, C, Neo, F
It should not disturb a sick baby	The neurological examination doesn’t disturb the baby. Unless you have to sit and turn around and put him prone and all of that, but the neurological examination of the baby that is sick should not include any of these	14, C, Neo, F
Aspects can be performed / observed at times of procedures	I do a lot of lines still. I see that as an opportunity really. How babies react to procedures tells you a lot about them and neurology is part of it… I see procedures as an opportunity to assess babies even better because you are spending a lot of time next to them	5, C, Neo, M
***Challenges to the neurological examination***		
Sedation	I certainly think [examining a sedated baby] is worth it. As long as you are aware… you document that they are on a medication that is going to affect the neurological examination. But you wouldn’t not do a respiratory examination because they’re intubated. You wouldn’t not do a cardio examination because they’re on cardiac medications. You’re still going to do those assessments. And I think people always would do those assessments. But for some reason they don’t in neurology because they’re on neurology medications	2, Tr, PNeurol, M
Cardiovascular instability	When they become unstable physiologically it becomes effectively impossible to safely do it	11, C, Paed, M
Time and developmental care philosophies	When you are in the neonatal unit the nurses… there’s always this thing in the nurses eyes, “don’t disturb the baby!”	18, Tr, Paed, F
Communicating results via telephone	If you ring… somebody rings you in the middle of the night, um, which happened last weekend… it’s really hard to get a sense of how people assess neurological status or degree of encephalopathy	5, C, Neo, M
Don’t actually know how to do a neurological examination	A lot of what we do is, you know, extrapolated from children at adult settings, where it’s just not really appropriate	7, C, Neo, M
	I think it’s a general reluctance and that’s not because people are lazy, I think it’s because people don’t know what they are doing. And so… people would rather go and do other things than do the neurology	8, Tr, Neo, F
	I think everybody is afraid of the neurological exam. Everyone is afraid of getting it wrong erm and for some reason it’s very daunting	21, Tr, Paed, F
Understanding what abnormal signs mean and interpreting the results	So, people can do the individual bits, but what they don’t know is, how to work out what that picture truly signifies. And I suspect we’re all doing that for Neurology	3, C, Neo, M
No agreed structure to the examination	The dedicated, sequential neurological assessment is lacking in neonatal set-up	16, C, Paed, M
Poor or no training	We don’t teach it in medical school very well. We don’t stress about it in the medical curriculum at all. So there are days and days of teaching about how to examine the upper limb and how to examine the lower limb in an adult. But there is very little teaching in the medical school about neurological examination in the newborn infant	9, C, Neo, F
	Doctors don’t get any dedicated neonatal neurology examination training	16, C, Paed, M
	There’s a lack of interest in the neurological assessment of children and babies. Full stop. And the training is grossly inadequate	10, C, Neo, M
	I think everybody, sort of, assumes that you can do it and that you will pick it up as you go along	19, Tr, Paed, F
Don’t know how to document it	I think we don’t have sometimes the words and the structures to document what we see in front of us	7, C, Neo, M
Consultants struggle too	I don’t think consultants do it very well	23, C, PNeurol, F
No modelling by consultants	Trainees just don’t see enough neurological examinations being done. I think that’s part of the issue	1, SG, PNeurol, F
Assessing competency	Sadly, I have not even done any CEXs for them about, you know, examinations	17, C, Paed, F
Wider problem involving all of paediatrics	I think to a degree it is the same across all ages in the neurological examination. But I think it’s… it’s more so exaggerated in neonates. Because I think people find the examination more difficult	2, Tr, PNeurol, M
	I think neurology, generally, if I go back, you know, years and years, I think it is probably the thing that people are the least comfortable with for whatever reason. I don’t think it’s specific to neonates. So, I think in paediatrics it’s the same: people don’t really examine the neurology properly	8, Tr, Neo, F
***Response to challenges: Avoidance***		
Avoidance or cursory examination	You’d always think, “Oh, this is not an emergency, I’ll let someone else do that or someone who kind of knows what they’re doing.”	1, SG, PNeurol, F
	One of my bug bears that I think that people often go with ‘AF normal, tone okay’. That’s not really a neurological examination. That is a box-ticking exercise	9, C, Neo, F
Legitimate challenges become excuses	So, because we don’t think hard enough about it, we use, for want of a better expression, excuses to not do the examination rather than think about… when, with another system like respiratory, we think about ‘oh what can I do?’, with neurology, we default to ‘I can’t do that’	9, C, Neo, F
	So similarly, um, we perhaps shouldn’t, you know, be using [the fact the baby is sick] as an excuse because obviously there can be findings there that determine how we manage this patient and whether the management will be different, or not	18, Tr, Paed, F
	It is almost a… a get out-of-jail card if they’re on medications that affect your nervous system because people will just say, “can’t assess neurology because… because they’re on such and such medication.”	2, Tr, PNeurol, M
	I don’t think the nurses would stop you if you wanted to assess the baby—that’s an excuse!	17, C, Paed, F
Delayed or missed diagnoses	There’s lots of, sort of, anecdotal stories, isn’t there, of babies who, you know, it’s only a week later that someone realises they’re not really moving their legs and they’ve got a spinal cord problem for example or, um, they’ve got a… I don’t know, they’ve got some focal signs that it would have been helpful or… you know, they should have had a scan earlier or something like that	1, SG, PNeurol, F
***Response to challenges: Solutions sought***		
Self-taught or trained abroad	Because my training was from a different, distant country, examination was… was drilled in. If you didn’t do an examination properly you were properly told off	6, C, Neo, M
	I read lots of things about it. And I tried it out on babies when it was needed	9, C, Neo, F
Introducing examination proformas into units	We have actually put this examination sheet on the network website for people to therefore look at it and try and make a better assessment of that	6, C, Neo, M
Using standardised examinations	Out of several examinations available, we summarised the HNNES (Hammersmith Neonatal Neurology Examination) for babies undergoing hypothermia	14, C, Neo, F
Improving training	Since I’ve joined here, err, I started doing 6 monthly erm neurological days study days and one of the topics which we do cover is the neonatal neurological examination	20, C, Neo, F
***Embedding new culture in unit***		
Low priority culture	It is just a self-perpetuating thing. You don’t do it. You don’t know why. And then you think there is no problem with that	6, C, Neo, M
	The problem is not the lack of a tool. It is one of the problems- but it is not the main problem. The main problem is the culture. The main problem is the way we are trained to think of the neurological examination being a ‘not important’ part of the newborn examination. And I think that is what needs to change	9, C, Neo, F
Culture of importance	I think part of it is about, um, you know, changing our culture, um, and the way we, sort of, approach neurological examination in general. Um, and that can be fun and nice, and quick. And it doesn’t need to be this absolute mountain that you have to climb every time	18, Tr, Paed, F
	If you work in a hospital where there is a perinatal hypoxia management protocol that mandates that the doctor has to go back and examine the baby, it’s done. If that’s not there, then very unusual, very unusual. It’s definitely an overlooked bit of the neurological examination	9, C, Neo, F

*Abbreviations: ABC* Airway, Breathing, Circulation, *Tr* Trainee, *SG* Staff Grade, *C* Consultant, *Paed* Paediatrics, *Neo* Neonatology, *PNeurol* Paediatric Neurology, *M* Male, *F* Female, *CEX* clinical evaluation exercise, a UK formative assessment of competency

In the low priority culture, decisions on whether to start hypothermia therapy in HIE were obvious without an in-depth assessment of the baby’s neurology. Clinicians were focussed on resuscitation, stabilisation, and time-critical procedures, such as securing venous access. The need to obtain competencies in procedures, and the perception that procedures were *“fun”*, meant trainees prioritised these above the neurological examination. Some interviewees did not think the neurological examination gave them useful information at all, “*Is me disturbing this child so much going to add to the clinical picture?”,* and this was seen particularly in interviewees who considered the neurological examination time consuming*.* Other interviewees noted that, once resuscitation and stabilisation had occurred, the neonate may have had sedative or paralysing medications, which rendered the neurological examination useless. When they felt unable to assess the neonate neurologically, interviewees relied on information from other sources, such as blood gases, cranial ultrasound, and amplitude integrated electroencephalography (aEEG).

Other interviewees considered the neurological assessment important. In the context of HIE, they acknowledged the need for *‘fire-fighting’*, i.e., focussing on emergency aspects of care, but felt the neurological assessment was important to justify decisions about hypothermia treatment and to provide a baseline against which change could be monitored. For these interviewees, the neurological examination was not time consuming, did not disturb a baby excessively, and interviewees thought aspects could be performed at the same time as procedures, such as assessing response to pain. In non-HIE cases, the neurological assessment was important to determine the neuroanatomical site of signs, form differential diagnoses, formulate management plans, and was described as *“fun”*.

The second subtheme outlined challenges to examination, including sedation, cardiovascular instability, negotiating arterial / venous catheters, ventilation, and finding a suitable time. The latter included disturbing the baby as little as possible by grouping tasks around the time of developmental cares. Other challenges related to understanding the findings of a neurological examination over the telephone, specifically discussions between units on the suitability of hypothermia therapy. The greatest challenge faced was that most interviewees did not know what to do to perform the examination or how to interpret the findings. There was no accepted structure to the examination, consultants and trainees had received no training in it during their career, they struggled to extrapolate adult-style examinations to the sick neonate, and they did not know how to document their findings. Trainees wanted more training, but the consultants themselves did not feel confident in the examination or communicating what they had done. Trainees rarely watched consultants examine and did not receive assessments on their technique. Some interviewees noted this was a problem in the whole of paediatric training and not just neonatal training.

The response to these challenges took two forms, forming a third subtheme. The first was avoidance, where the neurological examination was either not performed or a cursory examination was undertaken: the *“AF [anterior fontanelle] normal, tone okay”* phenomenon. Legitimate challenges to examination then became excuses, including projecting the need to coordinate examination with cares and procedures on nurses, who allegedly would not let doctors examine a baby. Others defended nursing staff from these accusations and noted the nurses allowed examinations if there was good communication on why it was needed at that time. Some health care professionals thought a neurological examination was pointless in a child who had received sedation, or could not be done owing to lines and ventilation. As a result of avoiding examination, interviewees gave examples of missed or delayed diagnoses.

A smaller proportion of interviewees did not accept these excuses and sought to overcome challenges. They would perform a limited examination, working around lines and equipment, and noting the importance of serial examinations in sedated babies to monitor change. They had typically received training outside the UK or had self-taught themselves, adapting their style over years. One had created proformas for the examination and introduced them into their unit or network to promote better examination and communication. Two interviewees worked in units with a focus on neurological care that used standardised examinations, such as the HNNE [13–15], and provided teaching for trainees. Whichever response to the challenges was adopted, this became the culture embedded in local practice.

### Theme 2: The practicalities of the neurological examination

Illustrative quotations are presented in Table 4. The first subtheme comprised attitudes towards different aspects of the neonatal neurological examination, which broadly fell into two categories: the achievable or important, and the impossible or unimportant.

**Table 4:** Illustrative quotations for Theme 2 – practicalities of the neurological examination in unwell term neonates

Subtheme	Quotation	Interviewee (No., Grade, Speciality, Gender)
***Attitudes towards different aspects of the neonatal neurological examination***		
*The achievable or important*		
Assessing the level of consciousness	Why assessing conscious level is important for us immediately after birth is making this decision: does the baby have an encephalopathy, and should they be cooled or not…. it can be quite subjective	3, C, Neo, M
	I think it’s fairly simple, isn’t it? It… it almost feels to me like common sense. You know?…How difficult can it be to differentiate between somebody who is completely normal, to somebody who is completely unconscious, and somebody who is sat in the middle?	6, C, Neo, M
	I think it’s really hard to assess conscious levels in babies other than, “are they awake? Are they asleep?” Um, I think definitely it would be good… like, in adults and in children, if you have this scale that you can, use as a tool to assess and to quantify, in a way	18, Tr, Paed, F
	I would never think of using a Glasgow Coma Score. I’ve never seen GCS written in a baby’s notes, term or preterm, and I’ve never heard anyone in the notes or over the phone discuss the GCS of the baby when describing their neurological status. No. It’s not something I would ever use	4, Tr, Neo, M
	I would probably use it, but use the modified, um, GCS but not, er… maybe subconsciously rather than absolutely consciously	15, Tr, PNeurol, F
	I think AVPU is nice that it’s, um… you know, it’s fairly obvious if a patient responds to pain, right? And it’s fairly obvious if they respond to voice, and it’s fairly obvious if they are awake. But because it’s fairly obvious, it means that it’s not that sensitive to subtle changes in the patient’s status	11, C, Paed, M
	I’ll tell you one of the things that has recently come up is… there is a dropped-baby guideline that is being set up nationally… and as part of that they would like us to use a modified Glasgow Coma Scale. Neonatologists think that Glasgow Coma Scales are meant for Paediatricians or adults or whatever be the case and it’s not for neonates… But you’ve now just popped this in front of me and say, “actually look at it and tell me: can you do this?” I’m thinking “Of course I can!”	6, C, Neo, M
Muscle tone and power	Tone is easy	5, C, Neo, M
	*[Distinguishing tone from power]* I think trainees get it confused all the time	3, C, Neo, M
	I think their first assessment of power would be “are they making antigravity movements?” as a baseline….People want to do their formal assessments of power, which in a neonate you can’t do	2, Tr, PNeurol, M
	The power is to say when they, um, when they kick their legs, kind of, against you, or you’re holding their arms and they’re trying to free themselves from you	18, Tr, Paed, F
	Muscle power again depends on the state of the child	23, C, PNeurol, F
Anterior fontanelle	Assessment of the anterior fontanelle is a relatively straightforward thing to do because we all do it very frequently	9, C, Neo, F
	I don’t know what I’m doing with it. I mean, like… people like to tell me that they can work out whether the baby is dehydrated but… you’ve got to be profoundly dehydrated before your anterior fontanelle goes in. I mean, I suppose I do… I do feel it	7, C, Neo, M
	So, they may be wearing a head-gear for the tube where you can’t assess the fontanelle or even the head circumference	12, C, PNeurol, M
Movements and posture	Presence of abnormal movements is again an observation—very heavily dependent on experience	9, C, Neo, F
	Quality of spontaneous movements is an important thing to look at. It is easy to look at. It helps a lot	6, C, Neo, M
	It is fairly customary for us to start with looking at the posture of the baby, which itself is a marker of neurological status	9, C, Neo, F
*The impossible or unimportant*		
Truncal tone	Truncal tone would be difficult to assess if they are lying down and I can’t lift them up	21, Tr, Paed, F
Primitive reflexes	Primitive reflexes may not be possible if they’re fragile and on a ventilator. You’re not going to be able to pick them up or do a Moro	12, C, PNeurol, M
	Nearly everyone who’s done more than a week on neonatal, of neonatal attachment, should know how to do and interpret a Moro	9, C, Neo, F
	What is the value of a Moro reflex? I think there is a little bit of a lack of knowledge	6, C, Neo, M
Cry	I suppose, assessing what the difference between a ‘cry to pain’ and a ‘moan to pain’, and so an irritable… what’s the difference between an ‘irritable cry’, a ‘cry to pain’’, and a moan to pain’? People might struggle with that	2, Tr, PNeurol, M
	I think a normal cry is easy to differentiate between an irritable cry. And moaning is easy. So cry and moaning, you can differentiate between these two. And an irritable cry you can tell. I think you can differentiate these two	6, C, Neo, M
Sensory levels	Even trying to determine a sensory level, you can. Not always the most reliable, but in some situations it’s very obvious what the sensory level is when you examine the baby	1, SG, PNeurol, F
Tendon reflexes	I think tendon reflexes are doable, it just takes a bit of practice and you’ve got to be consistently doing them fairly semi-regularly to continue with that	11, C, Paed, M
	I have to say, um er, definitely deep-tendon reflexes is not something that I am, er, confident at doing in a neonate	18, Tr, Paed, F
	Well I think because we don’t use tendon hammers in neonatal unit. I use stethoscopes, which is a bad way	5, C, Neo, M
Cranial nerves	Cranial nerve examinations: oh my God! No, I don’t think I’ve ever done that in a baby	7, C, Neo, M
	Sucking: yes; pupil response: absolutely; gag reflex: we don’t do much, but yes, if this could be done; …. facial expression: yes;.… eye movements, including nystagmus, ophthalmoplegia: yes; visual ability, fixing-and-following in neonate … becomes difficult—I don’t find it very easy; …. pupillary reflexes: yes	16, C, Paed, M
***Standardised neonatal neurological examinations***		
Positive views	Our unit is quite good because most of … we have quite good AHP cover and all our AHP’s are trained in various neurological assessment err including the Hammersmith. So, babies who are on HDU / SCBU invariably will get weekly Hammersmith and that’s chartered in the notes and we are able to see it	20, C, Neo, F
	I love the stick diagrams. I think they are fantastic. Um, and I love the way that they, um, they tell you how to do it	10, C, Neo, M
	If I was a paediatrician at a DGH who hadn’t done a lot of neonates, and I was faced with a newborn baby, it might be quite useful structure for me. It would give me something… it would remind me, kind of, what to do	7, C, Neo, M
Negative views	I’ve never seen, you know, the Hammersmith model printed out and put in the notes with tick boxes	4, Tr, Neo, M
	If they’re really sick and they’re tubed there are certain things you are not going to be able to do	19, Tr, Paed, F
	When I was a very junior doctor, we used to go and do, sort of, assessments of gestational age, erm, using those standardised scores. I can’t remember what the name of the forms were, we haven’t used it for so many years now	3, C, Neo, M
	It’s a very long examination and this is something that almost borders into ‘do you really need to do it?’. Because it is quite disturbing to the preterm infant, or even to the term infant	9, C, Neo, F
	It’s got to be simpler. I think the more complicated things perhaps would help research more, but it probably wouldn’t help practical day to day basis at all	23, C, PNeurol, F
	The challenge is people need to be trained to do it properly	9, C, Neo, F
	The writing is quite small for somebody with my eyesight	7, C, Neo, M
	It’s busy. It’s got a lot of stuff in it. I think people need to think about, if they are trying to revamp this, to try and make it something that is useful that is one page and not one, two, three, four, five	6, C, Neo, M

*Abbreviations*: *GCS* Glasgow Coma Scale, *AVPU* Alert, Respond to Verbal command, Pain, Unresponsive, *AHP* Allied Health Professionals, *HDU* High Dependency Unit, *SCBU* Special Care Baby Unit, *DGH* District General Hospital, *Tr* Trainee, *SG* Staff Grade, *C* Consultant, *Paed* Paediatrics, *Neo* Neonatology, *PNeurol* Paediatric Neurology, *M* Male, *F* Female

The assessment of neonatal conscious level was considered important and an area participants spoke about at length. In the context of perinatal HIE, the assessment of consciousness determined the degree of encephalopathy and suitability for therapeutic hypothermia. Some participants reported it was obvious if a neonate was alert or comatose and the decision about therapeutic hypothermia was easy. Other participants noted that some neonates fell into a *“grey zone”*and this was difficult to quantify. No interviewee used a formal consciousness score system, although some reported they used a broad method of categorisation similar to the AVPU system (Alert – responds to Voice – responds to Pain – Unresponsive) advocated in the Advanced Paediatric Life Support course [16]. The disadvantage of the AVPU scale was its insensitivity in detecting subtle changes over time.

No interviewee used the Glasgow Coma Scale (GCS). Participants thought the GCS was not developmentally appropriate, too detailed, and did not provide useful clinical information. When asked how they assessed consciousness in a neonate, participants subconsciously adopted the same categories as the GCS and made it developmentally appropriate. They were surprised, when faced with a chart showing the modified GCS for children, how much of the scale they adopted, but they did not formally score their findings. Without a score, interviewees found it hard to quantify the degree of consciousness, document it, and explain their findings to others. This subjectivity meant it was impossible to assess consciousness serially over time by different team members. Participants noted that nurses and parents were better at detecting subtle changes over time because of the consistency and regularity in who was assessing the baby and they could note when they were “quiet”. This suggests some quantification of consciousness is possible in neonates. After reviewing the GCS and its scoring, participants thought a modified score could capture what the nurses and parents were detecting instinctively, although they were unsure whether they would use it without evidence to show it identified deteriorating babies or provided prognostic information. A small proportion of participants were uncomfortable causing pain in babies during an assessment of consciousness, whilst others saw this as being an important component that could be performed at the same time as other routine painful procedures. Two interviewees noted there had been a recent recommendation for including serial monitoring of consciousness in neonates who had fallen or been dropped on the postnatal wards [17], and the development on a new neonatal coma scale would support this.

Determination of limb muscle tone was considered both achievable and important. The assessment of power was also thought important, although participants noted trainees experienced difficulties in differentiating tone from power and the Medical Research Council (MRC) muscle grading system [18] was inappropriate for neonates. Power was generally assessed instead by observing the presence or absence of antigravity movements and resistance to procedures and examinations. One participant discussed the relationship between conscious level and assessment of power, noting a neonate had to be alert to assess power. Participants felt the assessment of the fontanelle was achievable, but a small number questioned what useful information it gave; it was generally used to diagnose raised intracranial pressure and one participant objected to a “sunken” fontanelle being assumed to correlate with dehydration. Logistically, the fontanelle examination was difficult to perform where apparatus or hats were attached to the neonate’s head*.* Posture, quantity, and quality of spontaneous movements were also considered important, but assessing quality of movements was felt to require considerable experience. Head circumference, rooting, grasp, and plantar reflexes were also considered possible and important.

Features of the neurological examination that fell within the impossible or unimportant category included head lag and ventral suspension, primitive reflexes, such as the parachute reflex, the assessment of cry, which was either impossible in a ventilated neonate or too subjective, and examination of sensation outside the context of a spinal lesion. There was a strong culture that the Moro reflex was a vital component of the standard neonatal neurological examination, although no participant clarified what useful clinical information it gave. It was considered dangerous in an unwell / ventilated neonate. Participants’ opinions on deep tendon reflexes were divided. Some thought they were important, but examination was hampered by the lack of available tendon hammers and the practice of using a stethoscope instead, whilst others thought they were too hard to obtain and did not offer useful information. Participants’ answers about the cranial nerve examination were interesting: non-neurologist participants were resistant to performing the cranial nerve examination and thought it was too hard or did not provide useful information. However, when faced with individual components, such as pupillary responses, eye movements, facial expression, suck and gag, the same participants reported these aspects were important and easy to perform. Neurology participants saw the cranial nerve examination as both possible and important.

The second subtheme related to participants’ views on standardised neonatal neurological examinations, specifically the HNNE. The participants who worked in units with an interest in neonatal neurology used this tool and adapted it to the clinical condition of the baby. Others had experience of it in the past, such as during training to estimate the gestation of a preterm baby but did not use it routinely on their neonatal unit. Participants reported it was too long and repetitious, the relevance and importance of several signs were unclear, it required significant training, and it included aspects that were impractical in unwell neonates. Participants felt the pictures on the HNNE proforma were helpful, which enabled them to know how to perform the parts of the examination and to indicate results quickly by circling without the need for writing. However, participants thought the proforma was too cramped and busy.

### Theme 3: changing the culture

Illustrative quotations are shown in Table 5. A culture change was thought necessary: *“There should be a change in culture in the neonatal units until it becomes the norm.”* This theme outlined recommended steps to achieve this. The first subtheme was elucidation: convincing health care professionals the neurological examination could improve care. The second was development, summarised by one participant as “*I think it is about time we had some sort of good clinical examination”*. Participants thought a new simplified standardised neurological examination would improve the quality of neurological examination, communication of findings, documentation, objectivity, and monitoring of change over time. Any new examination needed to be quick, simple, feasible, and reliant mainly on observation. To aid with interpretation, interviewees recommended a schematic or flowchart. Views on scoring systems were mixed, with some considering they would help monitoring over time, and others feeling the focus would be on the score and not the meaning of the findings. One neurology consultant did not see the value of a new examination, although reflected that *“there’s always scope for improvement and one probably doesn’t realise it till it actually happens.”* probably because they felt their skills were good and did not see the neonatologists’ perspective. Once a suitable examination was created, interviewees recommended teaching courses and videos, with an emphasis on the neurology examination, in the paediatric under- and postgraduate curriculum, which formed the third subtheme. Assessments were thought important to demonstrate competency. The final subtheme was that interviewees thought the examination should be embedded in practice by consultants and through guidelines and research protocols.

**Table 5:** Illustrative quotations for Theme 3 – changing the culture

Subtheme	Quotation	Interviewee (No., Grade, Speciality, Gender)
***Elucidation***		
I think we need to raise awareness. And I think the only way to raise awareness is making sure that it doesn’t… it doesn’t feel like the neurology of a baby is unimportant		6, C, Neo, M
You will have resistance, of course, people have fixed practices on how they do things. I think a lot of it is whether you are able convince, because you’re talking to a group of people who look after children on a day in day out basis, isn’t it? And if you’re able to convince them that this is going to make sense for the child, then you will be able to push it forward		23, C, PNeurol, F
***Development***		
Appropriate examination	I think the only way you can do this is you try and make it more structured	6, C, Neo, M
	It needs to focus on the things that you can do in a real world not in a perfect world, because it’s never going to be a perfect world because they are sick	4, Tr, Neo, M
	If it’s slightly shorter, I would really welcome it. Then you would do it, maybe, over time to see progression or improvement or, you know, if there is any deterioration	17, C, Paed, F
	The nursing staff could actually give you a lot more information	15, Tr, PNeurol, F
	I think just encouraging people to think whilst they are watching. Just to observe and look	7, C, Neo, M
Proforma to improve documentation	I think something as a proforma in a neurological examination would be useful	15, Tr, PNeurol, F
	Put an actual checklist in the notes, rather than relying on freehand documentation. Because if you have something ready to print out, and then you just have to tick which part you have examined, or say, “this needs examining later.” Um, if it’s sort of standardised between units as well, that would be really useful	13, Tr, Paed, F
Aid to interpretation	The tool is not much use unless it then leads to a “this constellation equals this”	3, C, Neo, M
	I think there’s too much mystery and… you know, there’s some new methods coming up and new scores coming up and, er… so, I think it… I think it needs to be made more accessible to trainees and clinicians as a whole	10, C, Neo, M
	An algorithm, you know, that points you more towards peripheral muscle rather than central nervous-type aetiologies	7, C, Neo, M
Communication of results	It would be handy to be able… over the phone for the registrar, to be able to say, “I’ve done a…” you know, whatever score it turns out to be called, “and they score this.” And then I can say, “Ok. Well, where did they lose points?”	11, C, Paed, M
Scoring	In the ideal world, everything should have a score attached to it and we add up the scores and that tells us which category the baby goes into	9, C, Neo, F
	So, change over time is nice if you’ve got a score or a scale, isn’t it, to follow changes over time?	12, C, PNeurol, M
	I don’t know if they maybe hamper people’s ability to think through things and understand things. I wonder if people who have less of an interest or, if you’ve got a very junior person doing something, they still might at the end have absolutely no idea of what that means. Whereas, if you’re going through something less structured and less with a score, it might help you think a bit more	8, Tr, Neo, F
No need	I would say is I have never felt that there was a deficiency, erm so I think in using anything new, one has to feel that there is the need for that. So, one has to be very clear about where is the area of need. And erm this is just my general impression of what this study is about, erm, because I haven’t personally felt a particular need in that area. But all the best, I think, you know, there’s always scope for improvement and one probably doesn’t realise it till it actually happens	22, C, PNeurol, F
***Training***		
Teaching courses and videos	I think a course is something that would help people	6, C, Neo, M
	I think there’s an opportunity for the clinical examination to have videos of abnormal signs demonstrated and to have people having to interpret them	11, C, Paed, M
Induction	Making it sort of part of the induction, and you know, making it part of routine practice, then automatically will include it in the normal things that we know that we need to be aware of	22, C, PNeurol, F
Curriculum	It needs to be a part of training right from early on. I think the ideal way is the way anybody learns to examine any patient in medical school – it’s got to be down at that level	23, C, PNeurol, F
	More focus on it in the curriculum, particularly in the Tier 1 curriculum, because that’s where it starts	9, C, Neo, F
Assessment	Workplace assessments are a good way of assessing	9, C, Neo, F
	I think there should be more of neurological assessments for babies and children in the… in the RCPCH assessments	10, C, Neo, M
***Embed***		
Culture change	It has to be a culture change. It has to be, like, “everybody, from now on we’re doing this.”	17, C, Paed, F
Guidelines and protocols	I personally think, the neurological assessment, there is potential for improvement and it could be better if we have a dedicated protocol or guideline	16, C, Paed, M
	I think it would be helpful if there was a guideline, um, of which babies we need to look out for	13, Tr, Paed, F
Using research studies to implement clinical change	So as part of the study you had to have this neurological examination documented in a specific sheet. So, my way of bringing that examination into the department was to say, “well, we actually are including babies for this study and therefore we have to do this.”	6, C, Neo, M
Modelling	You, kind of, need to improve the confidence of the Consultants to then filter down to the juniors	1, SG, PNeurol, F
	I think that kind of comes from the top… from the trainee’s point of view it comes from the culture of the Consultant	12, C, PNeurol, M

*Abbreviations*: *Tr* Trainee, *SG* Staff Grade, *C* Consultant, *Paed* Paediatrics, *Neo* Neonatology, *PNeurol* Paediatric Neurology, *M* Male, *F* Female

## Discussion

In 2017, ST4-8 paediatric trainees in our region reported they felt confident in performing the neurological examination in neonates with HIE [4], and the data from our national survey showed comparable results. In comparison, our interviewees lacked confidence. At face value, this data appears at odds with each other, but the discrepancy can be explained. Two observations suggest the confidence levels in our preliminary data were likely suggestive of over-confidence: the high frequency trainees claimed they documented the neurological examination in the medical notes and the limited aspects of the examination they said they performed [4]. In this study, responders to the questionnaire also reported a detailed neurological examination was found in the medical notes of half of unwell neonates, which was also contrary to both the authors’ and interviewees’ experience. The paediatric neurology responders reported lower rates, probably reflecting differences in what they thought constituted a “high-quality examination”. Another observation was that standardised neurological examinations were rarely used by participants in either our questionnaire or interviews, with most adapting the neurological examination for older child or adult to the unwell neonate. However, interviewees frequently stated they did not know what aspects of the examination they should be doing, suggesting variation in what is done. In this situation, paediatricians can feel confident in their practice without being competent, especially if not trained, appraised, or challenged by those with more experience in neurological examination.

There was also a pervasive attitude that the neurological examination was unimportant and yielded little useful clinical information in unwell neonates, particularly amongst those trained in the UK. When the examination was seen through the prism of perinatal HIE, a detailed neurological examination did not add useful information when care was focussed on stabilisation. At these times, it does not matter what parts of the examination are done, or if it is cursory, clinicians can feel confident they have obtained all the information they need. However, when faced with an interviewer with neurological expertise and questions focussed on interpretation and aetiology of signs, confidence fell. Interviewees admitted trepidation in performing and interpreting the neurological examination in an unwell neonate, which correlated with the lower scores for the interpretation of signs in our questionnaires. Further evidence that reported levels of confidence were not genuine was the fear we found around certain aspects of the neurological examination, particularly the cranial nerve examination. In both the questionnaire and interviews, participants reported the cranial nerve examination was hard to perform but reported the individual aspects of it were “easy”. This suggests paediatricians see components of the neurological examination as part of a holistic assessment of the baby without considering their neuroanatomical significance. Thereafter, the concept of “formal” neurological examination in a neonate becomes intimidating, and a culture of avoidance results.

Fear and avoidance of neurological assessment amongst health care professionals is not a new observation. “Neurophobia”, where neurology is feared and perceived to be the most difficult clinical speciality, was first described in 1994 [19] and has since been found by others, including in the UK [20–25]. In paediatrics, this is likely to be compounded by additional developmental and behavioural challenges [1]. In unwell term neonates, these challenges are further magnified by the emergency nature of many conditions, cardiovascular instability, lines and intubation tubing, neonates’ even more limited developmental abilities, and the practice of considering consciousness only in broad categories [26, 27] or not at all. The latter may explain why interviewees noted the assessment of tone and power could be easily confused by trainees: health care professionals caring for older patients would not try to assess MRC grades of power in a comatose patient who is unable to perform the motor tasks asked of them, and so the assessment of power in neonates needs to be linked more closely to consciousness.

Neurophobia does not need be a part of neonatal care. Several interviewees had gained additional training outside the UK, where there was a more positive attitude and training towards the neurological examination. Some UK units had adapted standardised examinations to the unwell neonate. These interviewees valued the information it gave them, particularly during serial monitoring. Proactive training had led to a more positive culture on their unit. This leads to the question on whether standardised examinations, like the HNNE, should be introduced across all neonatal units. There was a lack of enthusiasm for this amongst interviewees, who thought the HNNE was not designed for unstable neonates, its focus was more directed towards detecting abnormality rather than interpretation of signs, it was too long and intimidating, and the proforma cramped and busy. Interviewees and questionnaire responders wanted a new, fit-for-purpose standardised neurological examination for unwell neonates.

A number of recommendations can be made from our data:

1. *Development of a neonatal consciousness score* – this should be developmentally appropriate, utilising the broad categories of the GCS, have a clear scoring structure, and be objective and useful in clinical practice
2. *Development of a standardised neurological examination for unwell term neonates* which is short, safe in ventilated neonates, includes only relevant components, and makes it clear which neuroanatomical site is being examined
3. *Creation of an interpretation aid* to make interpretation of signs, formulation of differential diagnoses and management plans easier
4. *Improved training and education* of all grades of medical staff on the neurological examination with elucidation of why it is useful
5. *Assessment of competencies* once new examination tools are introduced into clinical practice to ensure trainees know how to perform and interpret it
6. *Research* into the new tools to demonstrate their clinical utility and expand their use.

There are limitations to our study. These include that we cannot be sure the results reflect the full range of views amongst paediatricians. Certain attitudes may have led participants to complete the survey or interview, and the snowball technique may identify participants with similar attitudes. The results will also reflect the authors’ experiences to some degree, which is a well-known aspect of qualitative research. Similarly, paediatric training in other health care settings may be different than in the UK and our data may not be generalisable in different contexts.

In conclusion, although confidence levels of performing a neurological examination in an unwell neonate are reported to be high, this confidence does not appear to be genuine. Paediatricians do not know how to perform a high quality neurological examination in unwell neoantes or how to interpret the signs. There is a culture of neurophobia in UK paediatric services, and the examination has secondarily become unimportant and is avoided. Poor training has contributed to this phenomenon. A small number of units have sought solutions to these problems, adapted standardised examinations, and organised training, which has led to a more positive culture. However, our interviewees did not think the current standardised neurological examinations were fit for purpose. A change in the culture is desired, which would start by the development of simple standardised examinations of unwell neonates and a neonatal coma score, alongside training of why it is important, how to perform and interpret the results, and formal assessments of competency.

## Supplementary Information

**Additional file 1.** Neonatal neurological examination survey.

**Additional file 2.** The neonatal neurological examination study interview schedule.

## Data Availability

The data from the qualitative interviews are not publicly available to maintain confidentiality of centres and individuals, as per ethical approval. However, all reasonable requests for information will be provided on request to the corresponding author.

## Abbreviations

AF: Anterior fontanelle
aEEG: Amplitude integrated electroencephalography
ANNP: Advanced Neonatal Nurse Practitioners
AVPU: Alert – responds to Voice – responds to Pain – Unresponsive
BPNA: British Paediatric Neurology Association
GCS: Glasgow Coma Scale
HIE: Hypoxic Ischaemic Encephalopathy
HNNE: Hammersmith Neonatal Neurological Examination
MRC: Medical Research Council
QUAN: Quantitative data
QUAL: Qualitative data
ST: Specialist Trainee
