# Supplementary material for: Attitudes towards the neurological examination in an unwell neonate: a mixed methods approach
Source: BMC Pediatr. 2022 Sep 23;22:562. doi: 10.1186/s12887-022-03616-4 (PMC9502918; doi:10.1186/s12887-022-03616-4)
Supplement: Supplementary file 1 — Additional file 1. Neonatal neurological examination survey. [file 12887_2022_3616_MOESM1_ESM.pdf]

Neonatal Neurological Examination Survey

We are interested in how doctors of all grades and ANNPs feel about performing a neurological examination in an unwell neonate. Please consider completing this survey online at or send it back to us in the envelope provided.

Please pass of the form to colleagues / trainees to help us collect as many responses as possible.

Specialty

Grade

Unit (optional)

These questions relate to an **unwell neonate** who you suspect has a neurological disease, like encephalopathy or weakness. Imagine they are ventilated or have umbilical lines etc in situ.

1. How often do you perform a neurological examination in an unwell neonate?

0 = never; 3 = about half the time; 6 = all the time

0	1	2	3	4	5	6

2. How well do you think you do each of the following?

0 = not at all confident; 3 = somewhat confident; 6 = completely confident

	0	1	2	3	4	5	6
Performing a neonatal neurological examination							
Interpreting results of a neonatal neurological examination i.e. establishing whether normal or abnormal; determining anatomical site of any abnormality							
Using results of neonatal neurological examination to make a management plan							
Using results of neonatal neurological examination to formulate a prognosis							

3. How well do you think the trainees in your department perform the neonatal neurological examination in an unwell baby?

0 = not at all well; 3 = somewhat well; 6 = completely well

0	1	2	3	4	5	6

4. How often do you find a detailed, good quality neurological examination in the notes of a baby with a condition like encephalopathy or weakness?

0 = never; 3 = around half of the time; 6 = every time

0	1	2	3	4	5	6

5. What challenges do you face when doing a neurological examination in an unwell neonate?

6. Please look at these types of neonatal neurological examinations, and select which you use routinely and which you do not use? (Select all that apply)

	I have never used this neurological assessment in an unwell neonate	*I used this in the past, but I do not use it routinely now*	*I use this in specific cases only*	*I use this routinely in most neonates with neurological problems*
Classical paediatric neurology examination adapted for neonates				
Hammersmith Neonatal Neurological Examination				
Adapted (i.e. you add or omit certain bits) Hammersmith Neonatal Neurological Examination				
Amiel-Tison Neurologic Assessment				
Brazelton Neonatal Behavioural Assessment Scale				

7. How easy is it to assess the following aspects of the neurological examination of a sick neonate?

0 = not at all easy 3 = somewhat easy 6 = completely easy

	**0**	**1**	**2**	**3**	**4**	**5**	**6**
Assessment of conscious level							
Quantity of spontaneous movements							
Quality of movement / presence of abnormal movement							
Tone in limbs							
Truncal tone							
Muscle power							
Deep tendon reflexes							
Primitive reflexes							
Cranial nerve examination							
Anterior fontanelle							
Pupillary responses							
Visual ability e.g. fixing / following							
Eye movements incl nystagmus / ophthalmoplegia							
Facial expression							
Fundus examination							
Gag							
Suck							

8. Do you think a new standardised neurological examination specifically designed for unwell neonates to help you determine where in the neurological system a problem was would be useful?

Yes ☐ No ☐ Not sure ☐

9. Would you be willing to participate in an interview about your thoughts on the neonatal neurological examination (estimated time around 1 hour)?

Yes ☐ No ☐ Not sure ☐

If yes, please provide contact phone number or email:
