# Supplementary material for: Attitudes towards the neurological examination in an unwell neonate: a mixed methods approach
Source: BMC Pediatr. 2022 Sep 23;22:562. doi: 10.1186/s12887-022-03616-4 (PMC9502918; doi:10.1186/s12887-022-03616-4)
Supplement: Supplementary file 2 — Additional file 2. The neonatal neurological examination study interview schedule. [file 12887_2022_3616_MOESM2_ESM.pdf]

The Neonatal Neurological Examination Study
Interview Schedule

Things to do before the start of the interview:

- Introduce yourself and explain you are a paediatric clinical research fellow. You are learning more about how doctors feel when they examine a sick neonate neurologically, and how well they think they can interpret the results of the examination
- Ensure you are somewhere quiet with minimal distractions
- Check the interviewee is happy to continue with the project
- Ensure they know you will record them on the dictaphones
- Ask them to sign and initial (NOT X) the boxes on the consent form
- Start both Dictaphones

Opening question

Can you tell me about your experiences of examining a baby neurologically?

- What sort of conditions may these babies have?
- How do you feel about examining a sick baby neurologically? Why?

Questions on attitudes to the neurological examination

Are there any situations where you think you should have done a neurological examination of a neonate but didn't?

- If yes, what stopped you from examining the baby?
- How often does that happen?

I'm interested to hear your thoughts on your colleagues' / trainees' practice – do they examine all of the babies they should neurologically?

- If babies are always examined when they should be, refer to survey data:
    - We did a survey of UK paediatricians and it suggested 1/5 of unwell babies didn't get a neurological examination when they should have – why do you think this happens?

Generally speaking, **<u>how well</u>** do you think trainees / your peers examine babies neurologically?

- Sometimes important decisions are made based on a trainees' examination – such as whether a baby with HIE should be transferred for cooling or to choose investigations in a floppy baby. How much should we rely on this assessment to make decisions? Why?

If the baby is ventilated, cardiovascularly unstable, or with umbilical or arterial lines, does this affect the examination? How?

- Do you think there are any solutions to these problems with examining a baby who has lots of tubes, leads and venous lines in place?

Some people have told us that medications like sedation and paralysis affect whether they do a neurological examination. Do you think it is worth doing an examination whilst the baby is sedated? If not, when is the time that the first neurological examination should be performed? Does it always get done at that time?

Some paediatricians tell us the neurological examination is either not important or isn't prioritised over tasks like siting intravenous lines. Is this your experience?

- If it is not a priority, how often do you see people going back to do it at a later time?
- Our survey suggests that people perform a cardiovascular and respiratory examination but not the neurological examination. Is this your experience? Why do you think that happens?
- Some people say they find it best not to disturb the sick baby rather than do a neurological examination. What do you think about that statement? Is that your experience for the examination of other systems too?
- We have been told that some nurses caring for a sick neonate discourage doctors from performing examinations. Is that your experience? What are your thoughts on that?

Questions on specific aspects of the neurological examination

Please look at the list of parts of the neurological examination **[figure one]**

- Which parts do you find the hardest to assess? Why?
- How well do you think people distinguish tone from power in a neonate?

I'd like to talk about how we assess a neonates' level of consciousness. How do you assess this?

- Do you think this method is reliable?
- How well can this method be used by different members of the team to assess if a baby's conscious level is improving or deteriorating over time?
- The Glasgow Coma score **[Figure two]** is used in older children and adults. We have a sheet with the modified child GCS on. Have you ever used it in a neonate?
  - What are your thoughts on using the GCS in neonates? How could it be improved?

Documentation of the neurological examination

Our survey of UK paediatricians suggested that there was no detailed, high quality documentation of a neurological examination in the medical notes in around 3/4 of unwell babies with neurological problems. Does that fit with your experience?

- Why do you think we found that result?
- Could it reflect problems with documenting the examination?
- How can the documentation of the neonatal neurological examination be improved?

Please look at this proforma for a standardised neonatal neurological examination called the Hammersmith examination **[figure three]**. What experience do you have of this examination?

- If any, what is good about it? What is difficult?
- [Explain the examination and proforma, if needed, including the abnormal column and the different developmental ages]. Are there things that you like about the proforma? Are there things you don't like about the proforma?
- Would using a tool like this one improve:
  - Documentation of the examination?

- How standardised the examination is (i.e. done the same way different people)?
- Making sure everything was done that should be?

- How useful do you think it is to have the pictures on the proforma?
- Are there any parts of this examination that you think are really useful?
- Are there any parts of this examination that you think are not possible to do in sick neonates?
- Are there any parts of this examination that you think are not useful?

Interpretation of the neurological examination

I'd like to talk about how people interpret the findings of a neurological examination. For example, how well they tell if the baby is normal or not and decide if the cause of a baby's floppiness is because of a disorder affecting the brain, spine, nerves or muscles. What is your experience?

- Why do you think people find the **interpretation** of the neurological examination difficult?
- What can be done to make it easier to interpret the results and make lists of differential diagnoses?

Training

I'm interested in how people learn to perform a neurological examination in a newborn baby – what type of training did you receive?

- Do you think that training was sufficient?
- Do you think this should be a training priority?
- What could be done to improve training?

Designing a new neurological examination

We are working on designing a neurological examination of the sick neonate.

- What are the important parts of the examination that we should include?
- How do we ensure it is documented well in the notes?
- Are there any ways we can help doctors interpret the results?
- How do we make it easy to monitor the changes in a neonate neurologically over time?
- Can you tell us if there are any ways we can improve confidence of doctors and nurses using the examination, particularly if they are not doing it regularly?
- How can time/length of examination best be managed?

Closing questions

- If we had to make sure there were one or two things we should put in a new neurological examination, what would they be?
- Are there any other things you would like to talk about that we haven't discussed?

Figure one

Anterior fontanelle

Suck

Pupillary responses

Gag

Tone in limbs

Primitive reflexes

Quantity of spontaneous movements

Assessment of conscious level

Quality of movement / presence of abnormal movement

Truncal tone

Facial expression

Muscle power

Deep tendon reflexes

Eye movements including nystagmus / ophthalmoplegia

Visual ability e.g. fixing / following

Cranial nerve examination

Figure two: The Modified GCS For Children (BPNA 2001)

Pain should be made by pressing hard on the supra-orbital notch (beneath medial end of eyebrow) with your thumb, except for M4, which is tested by pressing hard on the flat nail surface with the barrel of a pencil.

Score the best response with unclear or asymmetrical. If in doubt repeat after 5 minutes.

Aspect to be examined	Criteria	Score
Eye opening	Spontaneous	4
	To voice	3
	To pain	2
	None	1
	Eyes closed (swelling / bandage)	C
Verbal	Alert, babbles, coos, words or sentences usual ability	5
	Less than usual / irritable cry	4
	Cries to pain	3
	Moans to pain	2
	No response to pain	1
	Intubated	T
Motor	Obeys commands, spontaneous movements	6
	Withdraws to touch	5
	Withdraws from nailbed pain	4
	Flexion to supraorbital pain	3
	Extension to supraorbital pain	2
	No response to supraorbital pain	1

Figure three: The Hammersmith proforma

Hammersmith Neonatal Neurological Examination CODE_________ D.O.E.______

NAME ________________________________ SEX___ RACE_____ D.O.B._______ AGE______ G.A.______ BW ______

Posture and tone

						STATE	ASYMM
**POSTURE** Infant supine. Look mainly at position of legs but also note arms. *Score predominant posture.*	arms & legs extended or very slightly flexed	Legs slightly flexed	legs well flexed but not adducted	legs well flexed & adducted near abdomen	abnormal posture: a) opisthotonus b) marked leg extension, strong arm flexion		
**ARM RECOIL** Take both hands, quickly extend arms parallel to the body, Count to three. Release. Repeat 3 times.	arms do not flex	arms flex slowly, not always; not completely	arms flex slowly; more completely	arms flex quickly and completely	arms difficult to extend; snap back forcefully		
**ARM TRACTION** Hold wrist and pull arm upwards. Note flexion at elbow and resistance while shoulder lifts off table. *Test each side separately.*	arms remain straight; no resistance felt R L	arms flex slightly or some resistance felt R L	arms flex well till shoulder lifts, then straighten R L	arms flex at approx 100° & maintained as shoulder lifts R L	flexion of arms <100°; maintained when body lifts up R L		
**LEG RECOIL** Take *both* ankles in one hand, flex hips + knees. Quickly extend. Release. Repeat 3 times.	No flexion	incomplete or variable flexion	complete but slow flexion	complete fast flexion	legs difficult to extend; snap back forcefully		
**LEG TRACTION** Grasp ankle and slowly pull leg upwards. Note flexion at knees and resistance as buttocks lift. *Test each side separately.*	legs straight - no resistance felt R L	legs flex slightly or some resistance felt R L	legs flex well till bottom lifts up R L	knee flexes remains flexed when bottom up R L	flexion stays when back+bottom up R L		
**POPLITEAL ANGLE** Fix knee on abdomen, extend leg by gentle pressure with index finger behind the ankle. Note angle at knee. *Test each side separately.*	180° R L	≈150° R L	≈110° R L	≈90° R L	<90° R L		
**HEAD CONTROL (1)** ***(extensor tone)*** Infant sitting upright. Encircle chest with both hands holding shoulders. Let head drop forward.	no attempt to raise head	infant tries: effort better felt than seen	raises head but drops forward or back	raises head: remains vertical; it may wobble			
**HEAD CONTROL (2)** ***(flexor tone)*** Infant sitting upright. Encircle chest with both hands holding shoulders. Let head drop backward.	no attempt to raise head	infant tries: effort better felt than seen	raises head but drops forward or back	raises head: remains vertical; it may wobble	head upright or extended; cannot be passively flexed		
**HEAD LAG** Pull infant towards sitting posture by traction on both wrists & support head slightly. Also note arm flexion.	head drops & stays back	tries to lift head but it drops back	able to lift head slightly	lifts head in line with body	head in front of body		
**VENTRAL SUSPENSION** Hold infant in ventral suspension. Observe back, flexion of limbs, and relation of head to trunk. If it looks different, DRAW.	back curved, head & limbs hang straight	back curved, head ↓, limbs slightly flexed	back slightly curved, limbs flexed	back straight, head in line, limbs flexed	back straight, head above body		

Tone patterns

**FLEXOR TONE (1)**<br>**(on traction: arm versus leg)**<br>Compare scores of arm traction with leg traction.		score for arm flexion less than leg flexion	score for arm flexion equal to leg flexion	score for arm flexion more than leg flexion but difference 1 column or less	score for arm flexion more than leg flexion but difference more than 1 column		
**FLEXOR TONE (2)**<br>**(arm versus leg)**<br>Posture in supine.			arms and legs flexed	strong arm flexion with strong leg extension<br>*intermittent*	strong arm flexion with strong leg extension<br>*continuous*		
**LEG EXTENSOR TONE**<br>Compare scores of leg traction and popliteal angle.		score for leg traction more than score for popliteal angle	score for leg traction equal to score for popliteal angle	score for leg traction less than score for popliteal angle, by 1 column only	score for leg traction less than score for popliteal angle, by more than 1 column		
**NECK EXTENSOR TONE (SITTING)**<br>Compare scores of head control 1 and 2.		score for head extension less than head flexion	score for head extension equal to head flexion	score for head extension more than head flexion. but difference 1 column or less	score for head extension more than head flexion but difference more than 1 column		
**INCREASED EXTENSOR TONE (HORIZONTAL)**<br>Compare scores of head lag and ventral suspension.		score for ventral suspension less than head lag	score for ventral suspension equal to head lag	score for ventral suspension more than head lag but difference 1 column or less	score for ventral suspension more than head lag but difference more than 1 column		

Reflexes

**TENDON REFLEX**<br>Test biceps, knee, and ankle jerks.	absent	felt, not seen	seen	"exaggerated"<br>(very brisk)	clonus		
**SUCK / GAG**<br>Little finger into mouth with pulp of finger upwards.	no gag / no suck	weak irregular suck only<br>No stripping	weak regular suck<br>Some stripping	strong suck:<br>(a) irregular<br>(b) regular<br>Good stripping	no suck<br>but strong clenching		
**PALMAR GRASP**<br>Put index finger into the hand and gently press palmar surface. Do not touch dorsal surface.<br>*Test each side separately.*	no response<br>R L	short, weak flexion of fingers<br>R L	strong flexion of fingers<br>R L	strong finger flexion, shoulder ↑<br>R L	very strong grasp; infant can be lifted off couch<br>R L		
**PLANTAR GRASP**<br>Press thumb on the sole below the toes.<br>*Test each side separately.*	no response<br>R L	partial plantar flexion of toes<br>R L	toes curve around the examiner's finger<br>R L				
**PLACING**<br>Lift infant in an upright position and stroke the dorsum of the foot against a protruding edge of a flat surface.<br>*Test each side separately.*	no response<br>R L	dorsiflexion of ankle only<br>R L	full placing response with flexion of hip and knee & placing sole on surface<br>R L				
**MORO REFLEX**<br>One hand supports infant's head in midline, the other the back. Raise infant to 45° and when infant is relaxed let head fall through 10°. Note if jerky.<br>Repeat 3 times.	no response, or opening of hands only	full abduction at shoulder and extension of the arms; no adduction<br>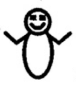	full abduction, but only delayed or partial adduction<br>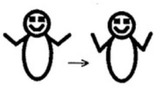	partial abduction at shoulder, and extension of arms followed by smooth adduction<br>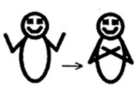	• minimal abduction or adduction<br>• no abduction or adduction; only forward extension of arms<br>• marked adduction only<br>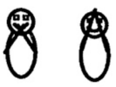<br>or		

Movements

**SPONTANEOUS MOVEMENT (quantity)** Watch infant lying supine.	no movement	sporadic and short isolated movements	frequent isolated movements	frequent generalized movements	continuous exaggerated movements		
**SPONTANEOUS MOVEMENT (quality)** Watch infant lying supine.	only stretches	stretches and random abrupt movements; some smooth movements	fluent movements but monotonous	fluent alternating movements of arms + legs; good variability	• cramped, synchronized; • mouthing • jerky or other abnormal movements		
**HEAD RAISING PRONE** Infant in prone, head in midline.	no response	infant rolls head over, chin not raised	infant raises chin, rolls head over	infant brings head and chin up	infant brings head up and keeps it up		

Abnormal signs/patterns

**ABNORMAL HAND OR TOE POSTURES**		hands open, toes straight most of the time	intermittent fisting or thumb adduction	continuous fisting or thumb adduction; index finger flexion, thumb opposition	continuous big toe extension or flexion of all toes		
**TREMOR**		no tremor, or tremor only when crying or only after Moro reflex	tremor occasionally when awake	frequent tremors when awake	continuous tremors		
**STARTLE**	no startle even to sudden noise	no spontaneous startle but reacts to sudden noise	2-3 spontaneous startles	more than 3 spontaneous startles	continuous startles		

Orientation and behaviour

**EYE APPEARANCES**	does not open eyes		full conjugated eye movements	*transient* • nystagmus • strabismus • roving eye movements • sunset sign	*persistent* • nystagmus • strabismus • roving eye movements abnormal pupils		
**AUDITORY ORIENTATION** Infant awake. Wrap infant. Hold rattle 10 to 15 cm from ear.	no reaction	auditory startle; brightens and stills; no true orientation	shifting of eyes, head might turn towards source	prolonged head turn to stimulus; search with eyes; smooth	turns head (jerkily, abruptly) & eyes towards noise every time		
**VISUAL ORIENTATION** Wrap infant, wake up with rattle if needed or rock gently. Note if baby can see and follow red ball (B) or target (T).	does not follow or focus on stimuli B T	stills, focuses, follows briefly to the side but loses stimuli B T	follows horizontally and vertically; no head turn B T	follows horizontally and vertically; turns head B T	follows in a circle B T		
**ALERTNESS** *Tested as response to visual stimuli* (B or T).	will not respond to stimuli	when awake, looks only briefly	when awake, looks at stimuli but loses them	keeps interest in stimuli	does not tire (hyper-reactive)		
**IRRITABILITY** In response to stimuli.	quiet all the time, not irritable to any stimuli	awakes, cries sometimes when handled	cries often when handled	cries always when handled	cries even when not handled		
**CONSOLABILITY** Ease to quiet infant.	not crying; consoling not needed	cries briefly; consoling not needed	cries; becomes quiet when talked to	cries; needs picking up to be consoled	cries; cannot be consoled		
**CRY**	no cry at all	whimpering cry only	cries to stimuli but normal pitch		High-pitched cry; often continuous		

SUMMARY OF EXAMINATION:

HEAD AND TRUNK TONE: LIMB TONE:

MOTILITY: REFLEXES:

ORIENTATION AND ALERTNESS: IRRITABILITY:

CONSOLABILITY: LIST DEVIANT SIGNS:
